# Eyewitness Identification: Live, Photo, and Video Lineups

**DOI:** 10.1037/law0000164

**Published:** 2018-08

**Authors:** Ryan J. Fitzgerald, Heather L. Price, Tim Valentine

**Affiliations:** 1 Department of Psychology, University of Portsmouth; 2 Department of Psychology, Thompson Rivers University; 3 Department of Psychology, Goldsmiths, University of London

**Keywords:** photo lineup, video lineup, live lineup, corporeal lineup, identity parade

## Abstract

The medium used to present lineup members for eyewitness identification varies according to the location of the criminal investigation. Although in some jurisdictions live lineups remain the default procedure, elsewhere this practice has been replaced with photo or video lineups. This divergence leads to two possibilities: Either some jurisdictions are not using the lineup medium that best facilitates accurate eyewitness identification or the lineup medium has no bearing on the accuracy of eyewitness identification. Photo and video lineups are the more practical options, but proponents of live lineups believe witnesses make better identification decisions when the lineup members are physically present. Here, the authors argue against this live superiority hypothesis. To be superior in practice, the benefits of live presentation would have to be substantial enough to overcome the inherent difficulties of organizing and administering a live lineup. The review of the literature suggests that even in experimental settings, where these difficulties can be minimized, it is not clear that live lineups are superior. The authors conclude that live lineups are rarely the best option in practice and encourage further research to establish which nonlive medium provides the best balance between probative value and practical utility.

Consider the eyewitness identification procedures in the following selection of robbery investigations. In *R v. Clothier* (2015), a female victim identified two robbers from a group of men standing in a line at Groblersdal Police Station. In *R v. Mongan* (2015) police visited a teenage victim at Royal Victoria Hospital, where he was recovering from a violent incident. After viewing video clips of men turning to the right and then the left, the youth identified the sixth lineup member with certainty. In *R v. Soderstrom* (2015), a female victim in Chilliwack viewed two photo lineups within hours of a home invasion. She identified one lineup member because of his facial features and another one because he was familiar from another context.

Why did the lineup members appear live in the first case, on video in the second one, and in photographs in the third? A reasonable person might conjecture that although live lineups are usually preferred because they afford the best view of the lineup members, in some circumstances less optimal procedures are acceptable. After all, a hospital would have been no place for a live lineup in *Mongan*, and it would have been challenging to recruit people for a live lineup within hours of the crime in *Soderstrom*. But in reality, the primary determinant of the identification procedures in these cases was the jurisdiction of investigation: Groblersdal is in South Africa, where live lineups are the convention; Royal Victoria Hospital is in Northern Ireland, where police use video lineups; and Chilliwack is in Canada, where photo lineups are the norm. The next question, then, is why does the lineup medium vary from one place to another?

There seems to be a conflict—or at least a perceived conflict—between choosing the lineup medium that best facilitates accurate eyewitness identification and choosing the lineup medium that is most practical to construct and administer. Unlike live lineups, which require the lineup members (and other relevant parties) to be physically present for the identification procedure, photo and video lineups can be conveniently constructed by recording an image of the suspect and then choosing fillers from a repository of similarly recorded images. Live lineups are nevertheless preferred in some jurisdictions, which seems to be the consequence of a *live superiority hypothesis*: The belief that live presentation of lineup members yields the best eyewitness identification outcomes. There are theoretical grounds to predict that an eyewitness would fare best at a live lineup, where the lineup members are observed in their entirety and can even be seen walking or talking. Compare this to a photo lineup, normally composed of static mugshots, and the notion of live superiority seems all the more plausible. Even video lineups, as conventionally practiced, only show the lineup members from the shoulders up, neutrally posed, turning from side to side. To make the right decision, a witness might need access to cues available only at a live lineup. Nevertheless, in spite of its appeal to intuition, we have reservations about the notion of live superiority.

We are confident that the live superiority hypothesis exists, but doubtful that it is true. Our review of five countries shows that all either once had or still have policies suggestive of live superiority. But our review of the research literature reveals that empirical tests of the hypothesis are scarce. And from the small corpus of experiments available to review, we find no strong indication that live presentation improves lineup performance. What we do find, however, are numerous factors that could compromise the reliability of eyewitness identification from live lineups.

## Identification Medium Practices and Policies

Perhaps the most glaring difference in eyewitness identification procedures around the world is the medium through which the lineup members are viewed. If people on the street were queried about their script for a lineup, they might describe a witness inspecting individuals in person, from either within the same room or behind a one-way mirror. Live lineups such as these are still routinely administered in some places, but elsewhere it has become standard practice for eyewitnesses to view lineup members as recorded images. Photo lineups, the most common procedure, comprise static physical or digital mugshots of the lineup members. A limited number of jurisdictions have also begun to use video lineups, which portray moving images of each lineup member.^1^ In this section, we provide a glimpse into the international variation in the use of live, photo, and video lineups.

### England and Wales

There was once a longstanding preference for live lineups in England and Wales. The London Met Police had been administering live lineups since at least 1860 (Devlin, 1976), and live lineups were consistently favored in Home Office guidance throughout the 20th century. In English courts, the longstanding tradition had been that witnesses could view photographs before a specific person was suspected of the offense, but that a suspect should be presented alongside fillers in a live lineup (e.g., *R v. Melany*, 1924; *R v. Wainwright*, 1925). In effect, photo viewings were permitted during the detection phase of an investigation (e.g., a mugbook search), but live lineups were expected during the evidentiary phase. Policy guidance, grounded in the live superiority hypothesis, made clear that live identifications were preferred over photo identifications, and that live lineups should have greater weight in court:

Photographs of suspects should never be shown to witnesses for the purpose of identification if circumstances allow of a personal identification. Even where a mistaken identification does not result, the fact that a witness has been shown a photograph of the suspect before his ability to identify him has been properly tested at an identification parade will considerably detract from the value of his evidence. (Item 18, Home Office, 1969)

In recent years, video lineups have become the preferred procedure in England and Wales. Police forces now have access to continually growing databases of videos depicting people who have volunteered to be recorded as fillers (Kemp, Pike, & Brace, 2001). The 15-s standardized video clips depict a single person first facing the camera, then turning their head to the right and left for profile views, and then facing the camera again for a final frontal pose. Current guidelines specify that a suspect should normally appear in a video lineup, but a live lineup may be administered if a video lineup would be impractical (Home Office, 2017). Although photographs may be presented at the detection phase, this is not advised at the evidentiary phase if the suspect is available for a video or live lineup.

### United States

In the United States, eyewitness identification policies have been conspicuously neutral on the lineup medium. American guidelines typically include separate provisions for live and photo lineups, with no hint at a preference between the two (American Bar Association, 2004; Technical Working Group for Eyewitness Evidence, 1999). Nevertheless, a clear favorite is evident in U.S. practice. In a nationally representative survey of U.S. police agencies, 94% reported use of photo lineups and only 21% reported use of live lineups (Police Executive Research Forum [PERF], 2013). This is possible because, contrary to in England and Wales, identifications from photo lineups are admissible in most U.S. courts (Wells & Seelau, 1995).

One exception is in New York, where a narrow reading of State legislation has kept photo lineup evidence out of the courtroom. Pursuant to New York Criminal Procedure Law, a “witness who has on a previous occasion identified such person may testify to such previous identification” (Sections 60.25, 60.30). The key phrase here is “identified such person” (Giordano, 2014), which New York courts have long considered distinct from identification of a person in a photograph (*People v. Caserta*, 1966; *People v. Cioffi*, 1956; *People v. Hagedorny*, 1947), resulting in the inadmissibility of photo lineup identifications at trial. To be clear, photo lineups are still an integral part of investigations in New York, where common practice is to first administer a photo lineup and then, if the case stands a chance of going to trial, to obtain a subsequent identification at a live lineup. Frustration with the continued exclusion of photo lineup evidence is apparent in *People v. Woolcock* (2005), where it was noted that although juries are not privy to identifications from “excellent” photo lineups the courts have been willing to admit identifications from “less than compelling” live lineups because of the acknowledged difficulty of finding fillers for the live procedure.

Legislative changes favoring photo lineups are currently underway in New York and have been successful elsewhere in the United States. Following a recommendation from the Municipal Police Training Council (2015) for an immediate change in policy, New York legislators included a provision in Bill A8157B that would permit photo lineups to be admissible at trial. The bill passed in April 2017. In Chicago, a city with a historical preference for live lineups, a shift to nonlive lineups was initiated through the passing of 725 ILCS 5/107A-2. The law, which went into effect in 2015, clarifies that there is no preference between photo and live lineups. The change was deemed necessary due to the practical conveniences of photo identification, but a spokesperson for the Cook County State’s Attorney office claimed that live lineups were nevertheless “a stronger method of identification” (Fusco & Novak, 2014).

At the national level, photo lineups are likely to remain the predominant medium. In 2017, the Department of Justice issued a memorandum with detailed instructions on the administration of eyewitness identification procedures (Yates, 2017). As with previous guidance, the memo contained no explicit instructions on the lineup medium. But in her instructions, (now former) Deputy Attorney General Sally Yates acknowledged that photo lineups are more common than live lineups because they can be organized more quickly and they do not require the suspect’s presence. As long as photo lineups continue to be admissible in U.S. courts, it seems only a matter of time before all police agencies in the country discontinue the use of live lineups and rid themselves of the hassle of finding fillers to physically attend the identification procedure. We reviewed guidelines from 10 states and none indicated a preference between live and photo lineups.^2^ Nor did any of the national guidelines, legislative acts, or model policies make reference to video lineups.

### Canada

Canadian eyewitness identification guidelines were once tilted in favor of live lineups. In a report prepared for the Law Reform Commission of Canada, Brooks (1983) considered the lineup medium in depth and expressed ambivalence about whether live or photo lineups should be preferred. Despite his assessment that photo lineups were more convenient, easier to control, and less anxiety-provoking for the witness, Brooks ultimately concluded that live lineups were the better option. Above all, his choice was motivated by a live superiority hypothesis: “the most important reason for preferring [live] lineups over photographic displays is that they appear to be a more accurate method of identification” (p. 104). At one point, Brooks warned that his assessment was based on limited information and should be considered tentative. However, in his recommendations, Brooks was unequivocal: “[live] lineups shall be held except in special circumstances” (p. 26) and “the use of photographs to identify criminal suspects is permissible only when a [live] lineup is impractical” (p. 30).

This recommendation notwithstanding, photo identification has become the most widely used lineup method in Canada. Surveys show that Canadian police agencies from across the country all use photo lineups (FTP Heads of Prosecutions Subcommittee on the Prevention of Wrongful Convictions, 2005, 2011), a practice enabled by the admissibility of photo lineup evidence in Canadian courts. The current standards for eyewitness identification in Canada were recommended at an inquiry into the wrongful conviction of Thomas Sophonow (Cory, 2001) and accepted by the Supreme Court of Canada shortly thereafter (*R v. Hibbert*, 2002). The Sophonow report included dozens of recommendations but steered clear of the identification medium by specifying separate guidelines for photo and live lineups, with no preference for one over the other (see also FTP Heads of Prosecutions Subcommittee on the Prevention of Wrongful Convictions, 2005, 2011). To our knowledge, video lineups have not been considered for use in Canada.

### Australia

Australia is unique in that live lineups are preferred in policy, but photo lineups are common in practice. The Evidence Act, 1995 states that lineups should be presented live, provided that (a) the suspect is in police custody at the time of the identification procedure and (b) the suspect does not waive their right to participate in a live lineup. In addition to a longstanding preference for live lineups by the High Court of Australia (*Alexander v. The Queen*, 1981; *Festa v. The Queen*, 2001), the priority given to live lineups in the Evidence Act, 1995 was reaffirmed by the Australian Law Reform Commission in 2006. The Act makes no reference to video lineups but advises that photo lineups should be inadmissible unless a suspect is unwilling to participate in a live lineup, the suspect’s appearance has changed since the offense, or it would not be “reasonable” to hold a live lineup. To assess whether it would be reasonable, the Act permits consideration of the availability of fillers, the severity of the offense, and the importance of the identification to the case. These contingencies may partly explain the divergence between policy and practice in Australia. Another factor is that suspects are explicitly instructed that they can refuse a live lineup and request a photo lineup instead (Australian Federal Police Practical Guide, 2011). But if the conditions are reasonable and the suspect is willing, the Act states that a live lineup should be held.

Although live lineups continue to be policy preference throughout much of Australia, there has been a shift to alternative methods in some states. In *Winmar v. the State of Western Australia* (2007), following a review of both case law and psychological literature, the Supreme Court of Western Australia objected to “any suggestion that the [photo lineup] process is inherently inferior to [a live lineup]” and recommended that a “trial judge is not required to direct a jury that [photo lineup] identification is unreliable and dangerous per se, or that it is inferior to other types of identification.” Subsequent attempts to use photo identification as a ground for appeal in Western Australia have failed (e.g., *Zanon v. the State of Western Australia*, 2016). In South Australia, legislators took measures one step further. Following passage of the Evidence (Identification Evidence) Amendment Act, 2013, South Australian judges “may not suggest that identification evidence obtained from an identity parade by any means other than by a physical line-up of persons is inherently or intrinsically less reliable than evidence obtained from an identity parade by such means” (Evidence Act, 1929 [2013 revision], 34AB). But live and nonlive identifications do not enjoy equality in all parts of Australia. In Victoria, the Evidence Act, 2008 maintains the preferential treatment for live lineups, and the Supreme Court of Victoria recently referred to photo lineups as the “second best mode of identification” (para 48, *Director of Public Prosecutions v. D. J. C.*, 2012).

### South Africa

Although photo lineup evidence has been admitted in South African courts, live lineups remain the common practice (Tredoux & Chiroro, 2005). In interviews with senior South African state prosecutors, one showed interest in alternative methods but explained that live lineups are favored by the courts (Rust & Tredoux, 1998). Indeed, consistent with Australian policy, South African judges have argued that photo lineups should not be used if the suspect has been arrested and is available for a live lineup (*S v. Moti*, 1998). The argument is not that photo lineup evidence should be excluded in all circumstances, but rather that live lineups are the better option and that evidence from photo lineups, if presented at trial, should be treated with caution and skepticism. The central concern in *Moti* was whether the procedural safeguards that have been built into the formal proceedings of a live lineup (e.g., unbiased instructions, independent administrator) would be as rigorously adhered to at an informal photo lineup. But fairness to the suspect may not be the only reason live lineups are preferred in South Africa. According to Rust and Tredoux, another senior state prosecutor likes them because they provide “more information and clues about the suspect’s behavior” (p. 201).

If the prospect of identifying a perpetrator from a live lineup sounds intimidating, no comfort would be gained upon learning the procedure used by some South African police to document the identification. The Criminal Procedure Act 51 of 1977 gives police officers the authority to present suspects for identification, but does not specify how they should conduct the procedure. Nevertheless, a set of 18 rules has emerged from case law and legal commentaries (Du Toit, De Jager, Paizes, Skeen, & Van Der Merwe, 1987; *S v. Tanatu*, 2004). Although many of the rules are shared by the other countries we reviewed, one represents a significant departure: Rule 18 states that witnesses should identify the culprit by tapping a lineup member on the shoulder, and that this act should be photographed (for a vivid description of the application of this procedure in a case, see Rust & Tredoux, 1998). This practice was once a requirement in South Africa, and for many years it was also recommended in England and Wales (Home Office, 1969). Although no longer required in either country, 60% of detectives from the South African province of Mpumalanga (*N* = 30) reported that witnesses should be instructed to tap a lineup member on the shoulder to make an identification (Mokonyama, 2010).

## Experimental Research

What can account for the variation in preferences for one medium over another? Why do the English choose to present lineup members on video, the Americans with photos, and the South Africans in the flesh? Experimental research (or lack thereof) may be partly to blame. If experimentation had revealed one medium to be consistently superior to the others, a more uniform approach might have taken hold. But in academic reviews of the lineup identification medium, the only consistency has been tentativeness in the conclusions.

We know of only three review papers that give substantial consideration to the identification medium and its effects. In the first, which exclusively focused on the lineup medium, the authors ultimately concluded that “based on available research, there is no reason to believe that live lineups, videotaped lineups, or photo arrays produce substantial differences in identification performance” (p. 181; Cutler, Berman, Penrod, & Fisher, 1994). In more recent discussions of the identification medium, the conclusions are no more definitive. Brewer and Palmer (2010) proposed that “no clear and empirically supported direction can be given” (p. 81) due to the small number and low quality of available studies. Clark, Moreland, and Rush (2015) reported that performance improves if lineups contain more information than if they contain less—a coarse comparison between video lineups and photo lineups, respectively—but warned that they “make this observation . . . with caution” (p. 152) and that “additional research is needed to examine how eyewitness identification outcomes vary across photo, live, and video lineups” (p. 152). Below, we provide an updated review of the experimental literature.

### Live Versus Photo/Video

Live lineups are a rarity in eyewitness identification experiments. Much like the trend toward nonlive procedures in criminal cases, eyewitness scientists have relied almost exclusively on photo lineups (though video lineups are becoming increasingly common, particularly in U.K. research). The paucity of experimental research presents a challenge for the live superiority hypothesis; even if every experimental result supported it, the evidence would not be compelling. And in the compilation of identification outcomes listed in Tables 1 and 2, not a single experiment lends robust evidence of live superiority.[Table-anchor tbl1][Table-anchor tbl2]

In the most recent review, Clark et al. (2015) found only three experiments that compared live and video procedures with lineups that did or did not contain the target participants were trying to identify. Composite measures of target-present and target-absent performance revealed a slight advantage for live over video lineups in two of the experiments (difference in proportion of correct responses ranged from 4% to 6%; Cutler, Fisher, & Chicvara, 1989; Kerstholt, Koster, & van Amelsvoort, 2004). The third experiment showed a trend in the opposite direction (another very small difference in percent correct: 2%; Cutler & Fisher, 1990), but Clark et al. warned against interpreting the data from this experiment because the witnessed event had two target actors and the lineups were always target-present for one actor and target-absent for the other.

Additional comparisons between video and live lineups can be found in the literature, but they are not particularly informative. A central concern with these additional experiments is that their lineups always included the target (Brace, Pike, Kemp, & Turner, 2009; Shepherd, Ellis, & Davies, 1982). Without a measure of choosing when the target is absent, it is unclear whether a higher correct identification rate is indicative of better decision-making or an indirect consequence of an increased willingness to make an identification, which would increase the chance of a lucky guess landing on the target. Even without this caveat, these experiments have been equivocal. Shepherd et al. reported that live presentation increased the correct identification rate compared to video presentation in one experiment but reported the opposite effect in another experiment (for similar results using a showup identification test, see Valentine, Davis, Memon, & Roberts, 2012). Brace et al. compared live and video lineups, but they were primarily interested in the effect of the procedures on witness anxiety. Different targets and lineup members were used for the live and video lineups, so the identification responses were not comparable (note also that correct identification rates were uniformly low: 0% and 9%, respectively).

As we move now to comparisons between live and photo lineups (see Table 2), the early research of Dent and Stephenson (1979) draws attention to the medium’s effect on the psychological state of the witness. In their first experiment, Dent and Stephenson observed signs of discomfort, self-consciousness, and embarrassment among participants as they inspected the row of men standing for a live lineup. Compared to those who saw a photo lineup instead, participants in the live condition made fewer correct identifications and spent less time looking at each lineup member. Similar reactions, if not more pronounced, were observed in their second experiment, with child witnesses. In the live lineup condition, children appeared “frightened” and were unwilling to look closely at the lineup members. Some were so upset they could not complete the task. Even convincing the children to walk up and down the lineup in the first place required some nudging. In light of these behavioral observations, it may be unsurprising that the odds of a correct identification for children who received a photo lineup were 3.03 (95% confidence interval [CI] [1.45, 6.25]) times the odds for those assigned to the live lineup. Dent and Stephenson explored procedures intended to make live lineups more bearable for children in a third experiment, but strong conclusions were precluded by a ceiling effect (descriptive statistics for each procedure were not reported). In their fourth experiment, this time with adults, correct identifications were highest for live lineups viewed through a screen, second-highest for photo lineups, and lowest for live lineups viewed without a screen.

One observation from Dent and Stephenson’s (1979) work is that participants seemed reluctant to make an identification while in the presence of the lineup members. Across three experiments, the rates of nonidentification were consistently higher for live lineups than for photo lineups. The only exception was when the live lineup was separated from the witness by a screen. In that experiment, the odds of a nonidentification for the live lineup with no screen were 4.14 (95% CI [1.80, 9.57]) times as great as the odds for the live lineup with a screen. Although the absence of target-absent lineups in Dent and Stephenson’s experiments precludes any firm conclusions about the identification medium’s effect on response bias, other studies that have included target-absent conditions are consistent with the suggestion that witnesses are less inclined to identify a lineup member if presented in person than if presented via photo or video (Cutler & Fisher, 1990; Kerstholt et al., 2004; Peters, 1991).

In summary, the experimental literature lends no empirical support for the live superiority hypothesis. If anything, the data converge on the conclusion that witnesses are less likely to identify any of the lineup members if they are observed in person than if they are viewed with videos or photos. Keep in mind, however, that the small number of empirical comparisons involving live lineups limits what can be inferred from this literature.

### Photo Versus Video

To begin our discussion of photo and video comparisons, the most recent review of the identification medium requires some unpacking. Using meta-analysis, Clark et al. (2015) found that increasing the information available in a lineup can improve identification outcomes. The meta-analysis summarized data from six experiments, three older (Cutler & Penrod, 1988; Cutler, Penrod, & Martens, 1987; O’Rourke, Penrod, Cutler, & Stuve, 1989) and three more recent (Darling, Valentine, & Memon, 2008; Kerstholt et al., 2004; Valentine, Darling, & Memon, 2007). All of the “less information” lineups included photos and all of the “more information” lineups included videos, but Clark and colleagues were careful to avoid labeling them all “photo lineups” and “video lineups” because of idiosyncrasies in the earlier work. For instance, in two experiments (Cutler & Penrod, 1988; Cutler et al., 1987) participants had access to photo images while they observed either video clips (more information) or another set of photo images (less information). The earlier experiments, including the one by O’Rourke et al., were further confounded by accompanying the video clips with voice samples and full-body views but not doing the same with the photo images. Because of these factors, the advantage Clark et al. reported for the lineups with more information cannot be squarely attributed to the use of video.

Contemporary experiments provide more straightforward comparisons between photo and video lineups, but even they do not isolate the identification medium’s effect. In research comparing U.K. videos with static photos, for example, the lineup members are always portrayed turning from left to right in the video condition, but only portrayed in frontal pose in the photo condition (Beresford & Blades, 2006; Darling et al., 2008; Havard, Memon, Clifford, & Gabbert, 2010; Seale-Carlisle & Mickes, 2016; Valentine et al., 2007). Thus, the video and photo lineups consistently differed in (a) the identification medium and (b) the visible angles of the face. Although a moving face is easier to recognize than the same number of frames presented as an array of static images (Lander, Christie, & Bruce, 1999), this confound nevertheless leaves open the possibility that including static profile views in the photo condition would eliminate any observed improvements for video lineups.

Confounds are pervasive in the lineup medium literature, perhaps for good reason. Rather than a single variable with different levels, each identification medium is a “constellation of variables” (Clark et al., 2015). In applied settings, an identification medium is inherently associated with factors beyond the medium itself. At a live lineup witnesses typically can view the lineup members in their entirety; at a photo or video lineup witnesses typically see only the head and shoulders. Live and video lineups can be dynamic; photo lineups are inherently static. Live and photo lineups can be presented sequentially or simultaneously; videos lineups are naturally sequential. Because of these natural co-occurrences, the ecological validity of an experiment may be increased by the inclusion of confounds.

Researchers must decide whether a gain in ecological validity is worth the loss in experimental control. To resolve this dilemma, imagine a pure test of the identification medium. The photo condition might display a frontal-view head shot of each lineup member, which could then be matched in the video and live conditions by arranging for only the heads of the lineup members to be visible and instructing them to face squarely forward and remain still. If the objective were to isolate the effect of the medium, this manipulation would seem to provide a reasonable method of achieving it. But would a null finding be enough to recommend that practitioners abandon video and live testing and opt for the more convenient method of photo testing? On the contrary, the findings would have little if any relevance to investigatory practices because it is the additional factors made possible through live and video lineups that underscores the appeal of these less practical medium types.

In a recent online study with photo and video lineups, Seale-Carlisle and Mickes (2016) deliberately confounded the comparison to explore differences between U.S. and U.K. procedures. In the U.K. procedure, nine videos depicting lineup members turning from left to right were presented sequentially. All videos were recorded by a U.K. police officer and adhered to PACE specifications, meaning each lasted 15 s. Also consistent with PACE, the lineup was shown twice before participants could respond (presentation duration depended on Internet connection speed; average = 6 min). In the U.S. procedure, six front-view still images were presented simultaneously and participants could respond immediately. Compared to those who received the U.K. procedure, participants who received the U.S. procedure were more likely to correctly identify the target if present (39% vs. 20%, *OR* = 2.56, 95% CI [1.96, 3.34]) and also more likely to correctly reject the lineup if the target was absent (45% vs. 30%, *OR* = 1.91, 95% CI [1.48, 2.46]). Seale-Carlisle and Mickes acknowledged that the many differences between the two procedures precluded causal explanations of the U.S. advantage, but pointed to two possibilities: (a) U.K. lineups were presented sequentially and (b) participants in the U.K. condition may have lost attention during the lineup presentation. We find the second explanation more compelling than the first.

It seems unlikely that simultaneous presentation alone would cause such a robust advantage for the U.S. condition. Sequential presentation is known to reduce choosing, which, compared to simultaneous presentation, tends to correspond with a reduction in correct identifications in target-present lineups and an increase in correct rejections of target-absent lineups (Clark, 2012; Palmer & Brewer, 2012). But the data reported by Seale-Carlisle and Mickes (2016) do not fit the typical pattern: collapsed across target-presence, choosing in the U.K. sequential condition was higher than in the U.S. simultaneous condition (70% vs. 62%, *OR* = 1.42, 95% CI [1.19, 1.69]).

The duration of the U.K. lineup procedure, combined with the use of online data collection, seems like a more plausible explanation for the poor outcomes in the U.K. condition. Seale-Carlisle and Mickes (2016) collected their data from undergraduate students, who have been reported to be less attentive than other populations in online studies (Hauser & Schwarz, 2016). Online and lab data collection has led to similar results in one previous comparison between simultaneous and sequential lineups (Mickes, Flowe, & Wixted, 2012). Note, however, in that experiment participants in both conditions could make a decision as soon as a stimulus was presented. In Seale-Carlisle and Mickes’s experiment, participants in the U.S. condition were able to respond to the lineup immediately, whereas participants in the U.K. condition were forced to view the entire lineup twice before they could respond. There is a sensible justification for this discrepancy: PACE guidelines require witnesses to view the entire lineup twice before making a decision and we know of no US guidelines with an equivalent requirement. Nevertheless, the length of the U.K. lineup procedure in this study (reportedly, ∼6 mins) may have been too long for undergraduate students to attend to the videos in an unsupervised environment. The question at hand is whether attention would be similarly lost by witnesses in real cases who (a) would have an incentive to identify the culprit and (b) would observe the videos under the supervision of a lineup administrator.

We suspect that at the very least, real U.K. lineups would not perform as poorly as the online U.K. lineups. Take, for example, the dismal correct identification rate of 20% for the U.K. online lineup (Seale-Carlisle & Mickes, 2016). This is substantially lower than suspect identification rates in field observations with real witnesses in the United Kingdom, which tend to hover around 40% (Horry, Memon, Wright, & Milne, 2012; Memon, Havard, Clifford, Gabbert, & Watt, 2011; Pike, Brace, & Kynan, 2002). Suspect identifications in real cases are not directly comparable to correct identifications in experiments because, among other reasons, the suspect’s guilt is only known in the latter. But if suspect identifications in the United Kingdom have any diagnostic value, the rate for guilty suspect identifications alone would be higher than the rate that aggregates guilty and innocent suspect identifications. The rejection rates in the online experiment are also worth noting: Participants were just as likely to incorrectly reject a U.K. target-present lineup (30%) as they were to correctly reject a U.K. target-absent lineup (30%). This seems consistent with the suggestion that the online participants were not paying sufficient attention while the U.K. lineup was presented and underscores the need for additional research to determine whether the attentional demands of the U.K. video lineup would cause similar problems when a lineup administrator is present to supervise the witness.

When administered in the laboratory, comparisons between U.K. video lineups and photo equivalents have yielded mixed results (see Table 3). Valentine et al. (2007) found a significant advantage in correct rejections of target-absent lineups for moving relative to static images, *OR* = 3.94, 95% CI [1.17, 13.27], and also found a smaller nonsignificant increase in correct identifications from target-present lineups favoring moving images, *OR* = 1.53, 95% CI [0.71, 3.32]. Darling et al. (2008) also found a trend favoring correct rejections of moving over still images in lineups with fillers matched to the culprit’s description, *OR* = 3.70, 95% CI [0.85, 16.67], but in lineups with fillers matched to the suspect’s appearance the trend in correct rejections was in the opposite direction (i.e., favoring still images), *OR* = 1.21, 95% CI [0.33, 4.51], and correct identifications in target-present lineups trended higher for still images regardless of how fillers were selected, *OR* = 1.41, 95% CI [0.57, 3.53]. In a study with child witnesses, Beresford and Blades (2006) found no consistent advantage favoring photo or video lineups. Havard et al. (2010), who also tested children, found general trends favoring video when the target was present and photo when the target was absent (and these trends were even further complicated by developmental differences). None of these experiments provides conclusive evidence of a superior medium.[Table-anchor tbl3]

Other lab experiments with video, but not the U.K. method of administration, have similarly provided no clear support for either photo or video lineups. In one of the earliest tests, participants who observed a live event identified the culprit from photo and video lineups at comparable rates, which were uniformly high—92% and 95%, respectively—and indicative of a ceiling effect (Shepherd et al., 1982). Another null result was reported by Cutler, Penrod, O’Rourke, and Martens (1986), who found no benefit of supplementing a photo lineup with videos that depicted the lineup members walking in and out of a room, *OR* = 1.06, 95% CI [0.67, 1.67]. Kerstholt et al. (2004) found a trend in correct identifications favoring photo lineups, *OR* = 1.80, 95% CI [0.73, 4.42], but also found a trend in correct rejections favoring video lineups, *OR* = 1.56, 95% CI [0.66, 3.70].

Contrary the lackluster results discussed so far, a few experiments have suggested an edge for video lineups. In addition to the previously mentioned experiment in which video lineups yielded an increase in correct rejections of target-absent lineups with no associated cost in target-present lineups (Valentine et al., 2007), our literature review led us to three additional experiments with results that favored video over photo lineups. In perhaps the first comparison between photo and video lineups, participants viewed a video containing a target person and then provided confidence ratings for lineup members presented as monochromatic photos, color photos, or monochromatic videos (Sussman, Sugarman, & Zavala, 1972). Of the monochromatic conditions, those who saw videos significantly outperformed those who saw photos, *d* = 1.08 (0.23, 1.94), *OR* = 7.09 (1.50, 33.55). Participants also performed better in the monochromatic video condition than in the color photo condition, though this difference was not significant, *d* = 0.70 (−0.12, 1.52), *OR* = 3.55 (0.80, 15.88). In another early experiment, Schiff, Banka, and de Bordes Galdi (1986) compared photo and video lineups that each contained 6 targets and 12 fillers. The video lineups, which displayed the lineup members turning in a rotating chair, led to greater discriminability compared with the photo lineups, *d* = 0.61 (0.09, 1.13), *OR* = 3.02 (1.18, 7.73), an effect that was larger when targets were encoded in a video event (video lineup *d*′ = 1.59, photo lineup *d*′ = 0.73) than in a slideshow event (video lineup *d*′ = 2.38, photo lineup *d*′ = 2.02). In a more conventional implementation of video and photo lineups, Cutler and Fisher (1990) reported a large, albeit nonsignificant, increase in correct rejections of target-absent lineups in a condition with videos that portrayed the members walking and talking (81%) compared with a photo condition that included an image of the full simultaneous lineup and individual close-ups of each lineup member (57%), *d* = 0.63 (−0.09, 1.35), *OR* = 3.15 (0.86, 11.60). The advantage in correct rejections came at no cost to correct identifications in the target-present lineups, which were trivially higher in the video condition (31% vs. 29%), *d* = 0.04 (−0.71, 0.62), *OR* = 1.11 (0.32, 3.92). One caveat in Cutler and Fisher’s experiment, however, is that the target-present and target-absent lineups contained different people and were not counterbalanced (Clark et al., 2015). Each of these three experiments also had small samples.

In our review of empirical comparisons between photo and video lineups, we found some indicators of a benefit for video lineups, but such experiments were exceptions rather than the rule. Further, none of the experiments favoring video lineups had the sample size needed for confidence in the findings. The only experiment with an impressive sample size favored the U.S. lineup condition, which used photos, but confounding factors obscure the cause of this advantage (Seale-Carlisle & Mickes, 2016). In sum, the empirical literature provides no compelling evidence in favor of either photo or video lineups.

## Practical and Theoretical Considerations

In the absence of clear empirical guidance on which medium best facilitates eyewitness identification, police might choose to adopt the most practical medium that the courts in their jurisdiction will admit as evidence. Live lineups, which require witnesses, suspects, fillers, legal representatives, and police personnel to all appear in the same place at the same time, are unquestionably the least practical option. When live lineups were required in England, one review of case reports showed that less than half of those scheduled came to fruition, with most cancellations attributed to suspects or witnesses not turning up (Pike et al., 2002). The inability to find suitable fillers was another reason for cancellation (Pike et al., 2002). This difficulty is implicitly acknowledged in guidelines for the US, where the minimum recommended number of fillers is five in photo lineups and only four in live lineups (Technical Working Group for Eyewitness Evidence, 1999). Live lineups also tend to come with a heftier price tag than photo or video lineups. Unless the fillers are prisoners or police officers, which raises another host of issues, remuneration is likely expected. The logistical nightmare of arranging and administering a live lineup would seem reason enough to put the practice to rest. In some jurisdictions, precisely this is happening. Among U.S. police agencies who do not use live lineups (17 of the 30 agencies interviewed), the primary reasons were inconvenience and lack of value compared to photo lineups (PERF, 2013).

From an investigative perspective, the appeal of photo and video lineups requires no stretch of the imagination. The ability to create and electronically store vast databases of photos and videos transforms the filler recruitment process from hunting for physical people who resemble the suspect (or the culprit’s description) to the mere act of browsing through a computer database filled with images. Once an image of the suspect has been obtained, the primary reason for live lineup cancellations—suspects not showing up—is no longer a concern. Witnesses are also more likely to appear if they do not have to be in the same proximity as the perpetrator. Photo or video lineups can also be presented with a computer, which could be used to deliver clear and consistent instructions, record the witness decision automatically, and visually represent response options that might not be obvious to the witness, such as “not here” or “not sure” (Brewer, 2011). Although an initial investment was required to develop the video identification infrastructure in England and Wales, changing from live to video lineups reduced the cost of each lineup by a factor of five (BBC News, 2003). Presumably, photo lineups are even less expensive.

In spite of their practicality, the use of nonlive lineups in some jurisdictions runs the risk that the evidence will be given less weight (or even excluded) at trial. Photo lineups are widely used in Canada and the United States because they are practical and judges in these jurisdictions have a history of accepting them as legitimate evidence. But in other jurisdictions, judges consider photo identification to be inferior to live identification. This preference for live lineups has been primarily supported by two overarching claims: (a) identifications from live lineups are more reliable than from nonlive lineups, and (b) live lineups are fairer than nonlive lineups. In the following, we review the merit of the arguments underlying these claims.

### Is Live Identification Inherently Superior to Photo and Video identification?

There are intuitive reasons to believe that, all else equal, live presentation would be the best method to facilitate an accurate eyewitness identification. Live lineups can elicit cues of height, weight, voice, and motion not typically available in photo and video lineups (Clark et al., 2015). At a live identification procedure, lineup members appear in their entirety and can be asked to walk, to talk, or to make facial expressions. Requests to act out a behavior may not always be met with compliance, and in many cases the types of cues possible only at a live procedure would be superfluous for identification. Still, it seems reasonable to predict that the additional information conveyed in a live lineup would help some witnesses identify the culprit. Those familiar with the organization and administration of a live lineup may counter that the logistical constraints of live procedures would negate any benefits afforded by the availability of additional cues. This is a point to which we will return. But in this section we set practicalities aside and consider the theoretical basis of the live superiority hypothesis.

If a crime has been witnessed live, benefits may come from an identification procedure that is also administered live. When the cognitive processes at encoding and retrieval overlap, transfer appropriate processing is theorized to occur (Morris, Bransford, & Franks, 1977). From this perspective, the processes engaged while witnessing a live event would be more likely to be reengaged at a live lineup than at a photo or video lineup. Matching the test medium with the event medium would also conform to the encoding specificity principle (Tulving & Thomson, 1973), which emphasizes the correspondence between the encoding and retrieval contexts. Study-test congruency effects in recognition of pictures and faces lend empirical support for these theoretical accounts (Buratto, Matthews, & Lamberts, 2009; Goldstein, Chance, Hoisington, & Buescher, 1982; Lander & Davies, 2007). Live lineups could increase the availability of cues and, in turn, the likelihood of retrieving the encoded details. Simply put, identification success should be enhanced by the availability of encoded details at test.

The availability of dynamic cues, in particular, may increase through live presentation of the lineup members. At a live criminal event, witnesses can encode dynamic motions from the perpetrator (e.g., facial expressions). Contrary to invariant structures (e.g., ethnicity), which are stable, dynamic motions are subject to change and less reliable as cues to recognition. The supplemental information hypothesis proposes that although recognition via invariant structures is generally preferred, dynamic motions can facilitate recognition when invariant structures are deemed uninformative (O’Toole, Roark, & Abdi, 2002). Thus, if all the lineup members possess the invariant structures that were encoded, the witness may benefit from the presence of dynamic motion cues. The value of such cues would be heightened for witnesses who have encoded a dynamic identity signature (Lander et al., 1999; Yovel & O’Toole, 2016); that is, movements that are idiosyncratic to the perpetrator. Although a relatively prolonged exposure to the target may be needed to detect and encode movements that are diagnostic to the target’s identity (Butcher & Lander, 2017; O’Toole et al., 2002), increasing familiarity at encoding does not always increase the benefits of movement at retrieval (Lander & Davies, 2007), which suggests rapid extraction of dynamic identity signatures may be possible.

Another potential benefit of live lineups is that the lineup members’ bodies are normally in view, whereas in photo and video lineups usually only the head and shoulders are visible. The most reliable cues for person identification are typically, but not always, located above the shoulders. Person-matching research indicates that removing the body from view leads to only a small decrease in identification accuracy, whereas removing the face causes a substantial decline in performance (e.g., Burton, Wilson, Cowan, & Bruce, 1999). Nevertheless, bodies with no face in view can be matched (e.g., O’Toole et al., 2011; Robbins & Coltheart, 2012) and recognized (Hahn, O’Toole, & Phillips, 2016) at levels well over chance expectancy. Further, when trying to match two images of people with similar faces and dissimilar bodies, matching whole-person or body-only images can be easier than matching faces (Rice, Phillips, Natu, An, & O’Toole, 2013). Taken together, these findings suggest supplementing face cues with information from the body could improve lineup identification accuracy.

Although the points discussed in this section may provide ammunition for believers in the live superiority hypothesis, we nevertheless remain skeptical of its application to lineups in practice. In addition to the absence of experimental data in support of live *lineup* superiority, there have been few experimental tests even on the broader issue of live *identification* superiority. The benefits of dynamic cues and whole-body views have been tested almost exclusively with videos and photos, which leads us to the more fundamental question of whether live testing is required to reap the theorized benefits we have discussed. If viewing the bodies of lineup members were found to improve lineup identifications, this would not provide direct support for the live superiority hypothesis. Whole person views tend to be available in live lineups and head-and-shoulder views tend to be available in photo and video lineups; however this is a convention, not a necessity. There is nothing inherent about photo or video lineups that would preclude whole person views. Although dynamic cues are not possible in photo lineups, they are certainly possible in video lineups. Indeed, the standard experimental approach to test for benefits of dynamic cues has been through comparing static photos and dynamic videos (Yovel & O’Toole, 2016). Thus, even when issues of fairness or practicality are not considered, the live superiority hypothesis has few legs to stand on. And when these factors are taken into account, the case for live lineups over other procedures falls apart.

### Are Live Lineups Fairer Than Video and Photo Lineups?

Live lineups have been frequently cited as the fairest identification procedure. Influential court cases and legal reviews have questioned the fairness of photo identifications on three grounds (*Alexander v. The Queen*, 1981; Brooks, 1983; Devlin, 1976): (a) the right to observe: a photo identification procedure denies the suspect of the opportunity to monitor the conditions of the identification procedure; (b) the rogues’ gallery effect: a photo identification procedure implies that the lineup members have criminal records; and (c) transference of familiarity: photo identifications interfere with subsequent identifications from live lineups. Contrary to these suggestions, we believe that photo and video lineups are at minimum as fair as live lineups (and more likely, fairer). Below, we explain why we do not consider these arguments compelling and argue that numerous characteristics of live lineups make them difficult to conduct fairly.

At first glance, the right to observe argument has intuitive appeal. A suspect who is present for the identification could conceivably make note of, or even prevent, elements of the procedure that could bias how the witness responds. This logic rings particularly true if the procedure is overtly biased; for example, a lineup with a suspect of different ethnic origin than that of the fillers. In this situation, most suspects would at least be aware that something is not right. A suspect would also be able to make decisions if present that would be more difficult or even impossible if absent. For instance, in many jurisdictions the suspect is permitted to choose their position in the lineup. Position effects have been demonstrated empirically (Clark & Davey, 2005; Palmer, Sauer, & Holt, 2017) and if the suspect’s position were left up to the administrators, one might question whether the position assigned to the suspect was fairly chosen. But is a suspect only able to observe if the lineup is administered live?

In certain jurisdictions and under certain conditions, the medium used to present a lineup has direct implications on the suspect’s ability to observe. In the United States, following a Supreme Court decision (*United States v. Ash*, 1973), suspects have no right to counsel at photo lineups. In a previous decision (*United States v. Wade*, 1967), out of concern for the risk of suggestion, the suspect’s right to counsel while participating in a live lineup had been established. But it was decided in *Ash* that this right does not extend to photo lineups. The distinction was justified on the grounds that legal representation is intended to protect suspects from disadvantages stemming from their lack of familiarity with the law or their inability to defend themselves against a professional prosecutor. Because the suspect is not present at photo lineups, these disadvantages were perceived as inapplicable. Although the majority opinion in *Ash* acknowledged that suggestion in photo lineups was still possible, it was considered far less likely than in a live lineup. And if suggestion did occur, it was argued that the suggestive element could be reconstructed at trial.

The dissenting justices argued that suggestion could just as easily occur during a photo lineup, an opinion now supported by empirical evidence (Clark, Brower, Rosenthal, Hicks, & Moreland, 2013; Greathouse & Kovera, 2009), but since *Ash,* photo lineups have been routinely administered in the U.S. without even informing the suspect or the suspect’s representative that the identification procedure has taken place. At a live procedure, the suspect is inherently present and one might reasonably expect the suspect’s legal representation also to be present. But the right to counsel at a live lineup in the US only applies if the suspect has been formally charged (*United States v. Wade*, 1967) and in practice only 11% of U.S. police agencies report taking measures to arrange for the suspect to have legal representation at all live lineup proceedings (PERF, 2013).

If the aim were to ensure the suspect has representation at the lineup, recommending that lineups be administered live seems a roundabout way of achieving it. Why not simply advise that suspects should have representation present during any lineup procedure? Identification procedures in England and Wales have been normally administered in the presence of the suspect’s solicitor not because of the lineup medium, but rather because Code D guidelines required it.^3^ The notion that the lineup medium influences the presence of counsel simply does not reflect the facts on the ground.

Live lineups do not ensure the critical aspects of the testing conditions will be observed. The only meaningful difference between lineup medium types from a right to observe standpoint is that the suspect is present at a live lineup. However, witnesses are commonly permitted to make the identification from behind a one-way screen. And even if the suspect is able to observe the identification, it would be naïve to expect a nonexpert to know about and detect the myriad of factors that could compromise the procedure. From our perspective, the right to observe would be best served by video-recording the lineup procedure—whatever the medium—and preserving the conduct of the identification proceeding (see also Kassin, 1998; Sporer, 1993; *Winmar v. The State of Western Australia*, 2007). Although recording all lineups may not have been feasible at the time when the right to observe was first argued, this is now less of an issue thanks to the widespread availability and reduced cost of video equipment.

The rogues’ gallery effect is another idea that may have been a legitimate concern in the past, but seems no longer relevant in the digital age. Rogues gallery presumes that if witnesses know that police possess a photograph of the suspect, they will infer that the suspect has a criminal record. But photographs can now be obtained in a variety of ways. Mugshots are still used in U.S. photo lineups, but so are driver’s license photographs (PERF, 2013). More to the point, it would not seem outlandish that the police would ask a suspect who has no criminal record to be photographed. In *Winmar*, the Supreme Court of Western Australia acknowledged the possibility of undue prejudice many years ago when witnesses were shown mugshots of prisoners with inmate numbers in view, but pointed out that with modern technology mugshots can be altered to remove such incriminating characteristics. Put simply, the rogues’ gallery can be avoided with easily implemented safeguards and the use of photographs in itself need not imply that the lineup members have criminal pasts.

The last of the three arguments rests on the assumption that live identifications are inherently superior to photo identifications. Transference of familiarity, which occurs when the memory of a person identified at a photo identification procedure replaces the memory of the criminal observed at the witnessed event, is a legitimate concern associated with repeated identification procedures (Steblay, Tix, & Benson, 2013). Not much is gained from a live lineup if a photo identification has already been conducted because at the second lineup it is never clear whether the suspect was remembered from the witnessed event or from the first lineup. If live identifications were more reliable than photo identifications, there could be an argument against administering a photo lineup because it would taint the witness’s memory and ruin the opportunity to obtain evidence via the more reliable, live procedure. Here lies the problem: the reliability of live over photo identification has not been established. Without clear evidence of live superiority, transference of familiarity has no relevance to the question of whether photo lineups are a suitable alternative to live lineups.

Having addressed the three main criticisms of nonlive procedures, it is worth taking a moment to consider what makes a lineup fair. We believe that a fair lineup procedure should minimize or ideally eliminate the opportunity for the witness to determine the suspect’s identity without relying on recognition memory. Lineups are a recognition task designed to enable witnesses to share information beyond what they have already stated in their verbal accounts. If something about the behaviors of the lineup members or the structure of the lineup provide a nonmemorial route to identification of the suspect, the lineup would be considered unfair. With this operational definition guiding our judgment of fairness, we contend that, if anything, photo and video lineups should normally be fairer than live lineups.

A central concern about the fairness of live lineups is that suspects are likely to emit cues and, contrary to nonlive procedures, there is no opportunity for a second take to prevent the witness from observing these cues. Suspects are likely to be anxious during a lineup procedure and research suggests they have difficulty hiding this from observers. In one study, participants judged the suspect to be the most anxious, most insecure, and most helpless-looking member of the lineup; further, another group of participants instructed to pick the suspect were successful far more often than would be expected by chance despite not having witnessed the crime (Fabian, Stadler, & Wetzels, 1996). The generalizability of these results may have been limited by the fact that all the fillers in the lineup were police officers; but in experimental research (Weigold & Wentura, 2004), even people who have not committed a crime are more likely to be identified if they were told they were the suspect (and were incentivized to avoid being selected) than if they were told they were a filler (with no such incentive). Further, to assist with lineup identifications, witnesses may rely on the lineup members’ emotional state or their appearance of guilt (Flowe & Humphries, 2011; Flowe, Klatt, & Colloff, 2014).

Even if the suspect is able to effectively monitor and control their behavior, their identity may be revealed by cues emitted from the fillers. At a live identification proceeding, fillers commonly know the identity of the suspect. All it would take is for the fillers to be waiting in one room before the identification procedure and for the suspect to be waiting in another. The only way to avoid this would be to have a separate room for each member of the lineup, which is not always possible (Pike et al., 2002). Further, if more than one witness is involved, lineup administrators may need to offer suspects the opportunity to change their position in the lineup prior to each witness identification (Home Office, 2017). Thus, it seems reasonable to assume that fillers in live lineups will often be (a) aware of the suspect’s identity and (b) able to communicate cues to the suspect’s identity. Examples of filler behaviors that could implicate the suspect include glancing at the suspect or standing farther away from the suspect than from the other lineup members.

With video and photo lineups, lineup administrators have greater control over the behaviors of suspects and fillers. If a filler or suspect emits a cue during a live lineup, there are limitations in what can be done to minimize the cue’s influence on the identification decision. Much as an obscenity heard during a live TV broadcast cannot be unheard, a hint to the suspect’s identity during a live lineup cannot be unseen. Although these types of cues are also possible with photo or live lineups, the person producing the image has the opportunity to ask the lineup member to try it again.

The risk of the administrator leaking cues to the eyewitness may also be heighted at a live lineup because simple methods of reducing administrator influence like computer-based administration are more difficult than with photo or video lineups. To prevent the possibility of influence on the witness (even unintentional), there have long been calls for lineups to be administered by someone who is blind to the suspect’s identity (Wells, 1988). Computer administration can reduce the possibility of influence even further by minimizing the social interaction between the administrator and witness or, in the case of self-administration, eliminating interaction altogether while the images are in view (Brewer, 2011; Kovera & Evelo, 2017).

The flexibility of live lineups could also lead to unanticipated situations with implications for fairness. At a live lineup in England and Wales, for example, Code D stipulates that if a witness requests to hear the lineup members speak, the administrator should first advise the witness to make a visual identification if possible; however, if the witness continues to request information about voice, the administrator can ask the lineup members to speak. In some cases, auditory cues may facilitate accurate identifications (Melara, DeWitt-Rickards, & O’Brien, 1989) and, indeed, some scholars have proposed including voice cues in lineups to supplement information from the face (Levi & Lindsay, 2001). But permitting the lineup members to speak without taking precautionary measures in advance could present a serious risk to a procedure’s fairness. For example, a suspect with a distinctive accent not shared by the other lineup members would stand out if they were all asked to speak (Wells, 2001).

The suitability of fillers is central to the fairness of a lineup. Although common practice in the field is to select fillers who resemble the suspect in physical appearance (Home Office, 2017; PERF, 2013), researchers have proposed that a fair lineup comprises members who all match the witness description of the culprit (Luus & Wells, 1991; Wells, Rydell, & Seelau, 1993). If a lineup member does not contain a feature from the description, a witness could eliminate this person because they do not correspond with what they recalled at the time of the description. But if all the lineup members possess each of the features from the description, the witness is forced to go beyond the information about the culprit they already provided and rely on recognition memory to make the identification. If a witness gives a very detailed description or the goal is to find fillers who closely match the suspect’s appearance, it could be difficult to find people who have each of the features in short notice for a live lineup. Constructing a lineup with implausible fillers has the effect of increasing suspect identifications, regardless of whether the suspect is guilty or innocent (Clark & Godfrey, 2009; Fitzgerald, Price, Oriet, & Charman, 2013; Flowe & Ebbesen, 2007; Lindsay & Wells, 1980). Whether trying to match fillers to the witness description or to the suspect’s appearance, the objective would be more easily met by selecting images from an electronic database than by recruiting locally for a live procedure.

Attempts to quantify lineup fairness have tended to favor nonlive over live lineups. Filler quality can be empirically tested using a mock-witness paradigm (Doob & Kirshenbaum, 1973). Although variations of the procedure have been developed (Mansour, Beaudry, Kalmet, Bertrand, & Lindsay, 2017), the standard practice is to provide a description of the culprit to participants who did not observe the crime and then to instruct these nonwitnesses to choose the lineup member most likely to be the suspect. If all lineup members match the description, the suspect should be chosen no more than expected by chance. In one application of this paradigm with real U.K. lineups (Valentine & Heaton, 1999), the suspect identification rate for live lineups (25%) was more than double chance expectancy (11%) and significantly greater than the suspect identification rate for video lineups (15%), which was higher, but not significantly higher, than chance expectancy (11%). In a subsequent study (Valentine, Harris, Piera, & Darling, 2003), fairness of U.K. video lineups was unaffected by whether the suspect was of White European or African Caribbean ethnicity. This was seen as a benefit because African Caribbean fillers were reportedly more difficult to recruit than White-European fillers for live lineups.

### Considerations of Real World Confounds

Previously we considered whether live identifications might be more reliable than video or photo identifications, all other factors held constant. It is important to be mindful, however, that external factors are not held constant in the field and that in practice the lineup medium is inherently confounded. Here we examine three factors associated with live lineups that have implications for identification accuracy.

First, live lineups are likely to increase witness stress and anxiety. Victims of violent offenses probably do not ever want to see the perpetrator again, but this is precisely what happens at a live lineup. Even witnesses who were not victimized may find live lineups stressful. In one experimental study, 76% of participants reported feeling nervous while taking part in a live lineup despite having been explicitly told that the incident they observed was staged (Brace et al., 2009). Beyond considerations of the witnesses’ well-being, stress may have implications for the identification response. In addition to the aforementioned conservative response pattern associated with live lineups in experiments (Dent & Stephenson, 1979), a survey of witnesses to real crimes who attended a live lineup revealed that witnesses who feared reprisals were less likely to identify the suspect (Ainsworth & King, 1988).

Although witnesses may feel threatened by the prospect of identifying the perpetrator irrespective of the medium, the thought of a live procedure seems to amplify such concerns. When live identifications were preferred in the United Kingdom, witness cancellations were more likely for male than female suspects and for suspects aged 16–40 than for suspects outside that age range, which may suggest witnesses were cancelling to avoid confrontation with stereotypically intimidating suspects (Pike et al., 2002). In a survey of 448 U.K. citizens (Dalton et al., 2014), 43% indicated lack of anonymity and/or the threat of suspect intimidation would discourage them from attending a lineup, and 44% reported that the main advantage of video lineups would be the reduced pressure on witnesses. This observation corresponds with a field study in which officers reported that 80% of witnesses at a video identification were calm (Memon et al., 2011).

The second factor, which also has clear implications for memory performance, is cue consistency. Photo and video images provide a record of the suspect at the time of arrest, which may help to increase the consistency between the cues encoded at the event and those available at retrieval. Suspects who are released from custody have the opportunity to make an intentional appearance change prior to appearing at a live lineup. If a perpetrator who was observed with a beard decided to shave for the lineup, the likelihood of identification at a live lineup would be reduced. If the delay between the arrest and the lineup is long enough, the suspect’s appearance may even change without any deliberate effort. It may be possible to mitigate the issue of appearance change with photo or video lineups by preserving the appearance of the suspect at the time of arrest. It may also be the case that an eyewitness description of the perpetrator is inconsistent with the appearance of a suspect at the time of arrest, but is consistent with another available photo or video. Whether to use the most recently available image or the one that corresponds with the witness description may need to be decided on a case-by-case basis. However, the point here is that with live lineups, such options would not be available.

Third, live lineups are particularly susceptible to long delays. When live lineups were the default procedure in England and Wales, 90% of police officers reported that delay was a common problem and almost 80% described the consequences of delay as “quite,” “very,” or “extremely” serious (Pike et al., 2002). Archival studies suggest that the majority of nonlive lineups occur within a month of the event, but this rarely happens for live lineups. Although drawing inferences from archival studies has drawbacks, the data in Table 4 are nevertheless consistent with the logical expectation that live lineups take longer to arrange than nonlive alternatives. Numerous characteristics of live lineups make them difficult to organize quickly: fillers must be recruited to appear in person; a time when all the relevant players are available must be scheduled; and everyone must show up. Timely identification procedures are desirable not only for the efficiency of the justice system, but also because reliability is increased if witnesses are tested when their memory of the perpetrator is fresh (Deffenbacher, Bornstein, McGorty, & Penrod, 2008; Palmer, Brewer, Weber, & Nagesh, 2013; Sauer, Brewer, Zweck, & Weber, 2010).[Table-anchor tbl4]

## Summary Conclusions

We can draw three main conclusions from this review. First, international practices and preferences regarding the identification medium are anything but uniform. Our review revealed more differences than similarities. Video lineups are required by English policy unless an alternative medium can be justified. Live lineups are given the most weight at trial in South Africa and throughout much of Australia, but these two countries nevertheless differ in practice: the traditional practice of live lineups continues in South Africa, whereas the practical appeal of photo lineups has led to their adoption in Australia, particularly for high-volume crimes. In Canada and the United States, the two countries most closely aligned, live lineups are permitted but photo lineups are the norm. Yet even within the United States, variability in practice across jurisdictions is evident.

Second, based on the current state of knowledge, the live superiority hypothesis at present is merely a belief—not a fact. The experimental literature provides no clear direction on which medium, if any, is inherently better than the others. Ignoring the obvious practical constraints of organizing a live lineup, we considered whether live identification tests would be superior to nonlive identification tests if all external factors could be neutralized. In spite of our efforts to be as charitable as possible to the live superiority hypothesis, we found little reason to support it. The empirical evidence is inconclusive, and most of the theoretical mechanisms that could be considered consistent with live lineup superiority, such as availability of body and motion cues, could be incorporated into modifications of current video lineup practices. For example, one might argue that a live advantage in the empirical literature has been missed because researchers tend to use short exposure durations and longer durations would be needed for witnesses to encode a perpetrator’s dynamic identity signature. But this line of argument would only explain why we have not found an advantage of live over photo lineups. If a video lineup were constructed to portray the same information as a live lineup, the detection of a dynamic identity signature should be no less likely than if the lineup members are physically present.

Third, a policy preference for live lineups is untenable, due to (a) the lack of empirical support for the live superiority hypothesis; (b) the difficulties of administering a fair live lineup; and (c) the inherent practical advantages of nonlive procedures. Photo and video lineups are more practical, fairer, and seem to be no less reliable than live lineups. Live lineups are hard to organize and difficult to control. The inability to recruit suitable fillers or to prevent any of the lineup players from emitting unwanted cues could easily compromise the fairness of a live lineup. And the proposed safeguards that have been associated with live lineups (right to observe, rogues’ gallery, and transference of familiarity) do not hold up to scrutiny. Contrary to the live superiority hypothesis, we identified several real-world confounds associated with live lineups that could reduce their reliability.

We recommend against live lineups, but believe more evidence is needed before a preference between photo and video lineups can be established. Photo lineups are the most practical option, but the availability of dynamic information in video lineups may improve identification outcomes. More research is needed to determine whether video lineups lead to outcome benefits that justify their practical costs.

One avenue for future research would be to modify existing video techniques to maximize their potential benefits. The type of head-and-shoulders clips that have been adopted in the United Kingdom may not be exploiting all the advantages that video lineups have to offer. There have long been calls to test video lineups that show the entire body and provide additional cues of gait or voice (Shapiro & Penrod, 1986; Slater, 1994). Although these factors were once the focus of programmatic studies (Cutler et al., 1994), video quality has significantly improved since the completion of that work. Virtual or augmented reality may be another technological advance with a potential application for lineups. Although the realism of immersive environments could be problematic for victims of traumatic events, some witnesses could benefit from the increased viewing angles and physical context reinstatement that would be possible (Bailenson et al., 2008). Another consideration is whether ethnicity plays any role in lineup medium effects. For example, image quality and the availability of dynamic cues associated with a medium may be more critical when the witness and lineup members have different ethnic backgrounds (Sporer, 2001).

Until the science advances and clear superiority for one medium is established, we expect international variation in identification medium practices to continue. Photo and video lineups are unequivocally more practical than live lineups, but these nonlive techniques are up against the intuitive appeal of live lineups. In the absence of empirical falsification of the live superiority hypothesis, its proponents may be unwilling to sacrifice what they perceive as greater probative value in exchange for practical gain. This makes it imperative for eyewitness scientists to bring the identification medium back into the spotlight and strengthen the empirical foundation for a recommendation that will encourage all jurisdictions to adopt the medium that best facilitates eyewitness identification.

## Figures and Tables

**Table 1 tbl1:** Comparisons Between Live and Video Lineups

Target, outcome, and study	Event	Live			Video			Effect size and CIs			Test of null	
		Rate	Type	*n*	Rate	Type	*n*	*OR*	LL	UL	*z*	*p*
Present												
Hit												
Cutler and Fisher (1990)	Live	.23	Sim	26	.31	Sim	26	1.52	.44	5.26	.65	.52
Cutler, Fisher, and Chicvara (1989)	Live	.65	Sim	17	.69	Sim	16	1.20	.28	5.26	.24	.81
Kerstholt, Koster, and van Amelsvoort (2004)	Live	.69	Sim	58	.63	Seq	48	1.31	.58	2.93	.65	.52
Shepherd, Ellis, and Davies (1982), Exp. 1	Live	.67	Sim	12	.55	Sim	20	1.66	.37	7.38	.67	.50
Shepherd et al. (1982), Exp. 4	Live	.89	Sim	19	.95	Sim	20	2.33	.20	25.00	.68	.50
	Video	.44	Sim	18	.75	Sim	13	3.85	.80	16.67	1.68	.09
	BW Photo	.50	Sim	16	.85	Sim	15	5.56	1.01	33.33	1.97	.05
	Color Photo	.79	Sim	14	.53	Sim	13	3.34	.62	18.00	1.40	.16
Filler												
Cutler and Fisher (1990)	Live	.12	Sim	26	.12	Sim	26	1.00	.19	5.33	.00	1.00
Cutler et al. (1989)	Live	.06	Sim	17	.00	Sim	16	3.04	.12	79.88	.67	.50
Shepherd et al. (1982), Exp. 1	Live	.17	Sim	12	.20	Sim	20	1.22	.19	7.69	.21	.83
No ID												
Cutler and Fisher (1990)	Live	.65	Sim	26	.58	Sim	26	1.34	.44	4.12	.52	.60
Cutler et al. (1989)	Live	.29	Sim	17	.31	Sim	16	1.10	.25	5.00	.13	.90
Shepherd et al. (1982), Exp. 1	Live	.17	Sim	12	.25	Sim	20	1.64	.27	10.00	.53	.60
Absent												
No ID												
Cutler and Fisher (1990)	Live	.85	Sim	26	.81	Sim	26	1.33	.31	5.70	.38	.70
Cutler et al. (1989)	Live	.89	Sim	8	.63	Sim	5	4.75	.27	83.28	1.07	.29
Kerstholt et al. (2004)	Live	.43	Sim	58	.61	Seq	49	2.08	.95	4.55	1.85	.06
Both												
Choosing												
Cutler and Fisher (1990)	Live	.25	Sim	26	.31	Sim	26	1.35	.40	4.55	.48	.63
Cutler et al. (1989)	Live	.50	Sim	25	.58	Sim	21	1.39	.43	4.35	.54	.59
Shepherd et al. (1982), Exp. 1	Live	.83	Sim	12	.75	Sim	20	1.63	.27	9.99	.53	.60
*Note*. *OR* = odds ratio; CI = confidence interval; LL = lower limit 95% CI; UL = upper limit 95% CI; Sim = simultaneous presentation; Seq = sequential presentation; BW = black and white (monochrome); ID = identification. Choosing rates are collapsed across target-present and target-absent or (if no target-absent lineups were used) target-present only. To facilitate comparisons, all *OR*s below 1.00 and associated CIs are converted to their inverse.												

**Table 2 tbl2:** Comparisons Between Live and Photo Lineups

Target, outcome, and study	Event	Condition	Live			Photo			Effect size and CIs			Test of null	
			Rate	Type	*n*	Rate	Type	*n*	*OR*	LL	UL	*z*	*p*
Present													
Hit													
Cutler and Fisher (1990)	Live	—	.23	Sim	26	.29	Sim	21	1.37	.37	5.00	.47	.64
Dent and Stephenson (1979), Exp. 1	Video	—	.14	Sim	35	.29	Seq	35	2.50	.75	8.33	1.50	.13
Dent and Stephenson (1979), Exp. 2	Live	—	.12	Sim	98	.29	Seq	124	3.03	1.45	6.25	2.98	<.01
Dent and Stephenson (1979), Exp. 4	Live	Live with Screen	.40	Sim	50	.30	Seq	50	1.56	.68	3.56	1.05	.30
		No screen	.18	Sim	50	.30	Seq	50	1.96	.76	5.00	1.39	.16
Egan, Pittner, and Goldstein (1977)	Live	—	.98	Sim	40	.85	Sim	46	8.65	.82	91.29	1.79	.07
Kerstholt et al. (2004)	Live	—	.69	Sim	58	.75	Both	44	1.35	.56	3.23	.66	.51
Peters (1991)	Live	Crime	.33	Sim	12	.75	Sim	12	6.25	1.03	33.33	1.99	.05
		No crime	.75	Sim	12	.83	Sim	12	1.64	.22	12.50	.48	.63
Shepherd et al. (1982), Exp. 4	Live	BW photo test	.89	Sim	19	.81	Sim	13	1.90	.26	13.97	.63	.53
		Color photo test	.89	Sim	19	.92	Sim	15	1.43	.13	14.29	.29	.77
	Video	BW photo test	.44	Sim	18	.85	Sim	13	7.14	1.20	50.00	2.17	.03
		Color photo test	.44	Sim	18	.53	Sim	15	1.43	.36	5.56	.51	.61
	BW Photo	BW photo test	.50	Sim	16	.87	Sim	15	6.67	1.11	33.33	2.08	.04
		Color photo test	.50	Sim	16	.65	Sim	17	1.85	.44	7.69	.84	.40
	Color Photo	BW photo test	.79	Sim	14	.62	Sim	13	2.31	.46	11.61	1.01	.31
		Color photo test	.79	Sim	14	.69	Sim	13	1.69	.30	9.65	.59	.56
Sporer (1991)	Live	Sim vs. seq	.80	Sim	15	.62	Seq	13	2.45	.45	13.28	1.04	.30
		Seq vs. seq	.64	Seq	11	.62	Seq	13	1.09	.21	5.76	.10	.92
Filler													
Cutler and Fisher (1990)	Live	—	.12	Sim	26	.10	Sim	21	1.23	.19	7.82	.22	.83
Dent and Stephenson (1979), Exp. 1	Video	—	.14	Sim	35	.20	Seq	35	1.54	.43	5.56	.67	.51
Dent and Stephenson (1979), Exp. 2	Live	—	.34	Sim	98	.32	Seq	124	1.09	.62	1.92	.31	.75
Dent and Stephenson (1979), Exp. 4	Live	Live with screen	.30	Sim	50	.20	Seq	50	1.71	.68	4.30	1.15	.25
		No screen	.18	Sim	50	.20	Seq	50	1.14	.42	3.13	.25	.80
Peters (1991)	Live	Crime	.08	Sim	12	.17	Sim	12	2.38	.18	33.33	.65	.51
		No crime	.08	Sim	12	.17	Sim	12	2.38	.18	33.33	.65	.51
Sporer (1991)	Live	Sim vs. seq	.00	Sim	15	.15	Seq	13	6.67	.29	100.00	1.18	.24
		Seq vs. seq	.18	Seq	11	.15	Seq	13	1.24	.14	10.83	.20	.84
No ID													
Cutler and Fisher (1990)	Live	—	.65	Sim	26	.62	Sim	21	1.14	.34	3.76	.21	.83
Dent and Stephenson (1979), Exp. 1	Video	—	.71	Sim	35	.51	Seq	35	2.35	.88	6.31	1.70	.09
Dent and Stephenson (1979), Exp. 2	Live	—	.54	Sim	98	.39	Seq	124	1.84	1.07	3.14	2.22	.03
Dent and Stephenson (1979), Exp. 4	Live	Live with screen	.30	Sim	50	.50	Seq	50	2.33	1.03	5.26	2.02	.04
		No screen	.64	Sim	50	.50	Seq	50	1.78	.80	3.96	1.41	.16
Peters (1991)	Live	Crime	.58	Sim	12	.08	Sim	12	15.88	1.47	171.56	2.28	.02
		No crime	.17	Sim	12	.00	Sim	12	6.07	.26	140.49	1.13	.26
Sporer (1991)	Live	Sim vs. seq	.27	Sim	15	.15	Seq	13	2.10	.31	14.04	.76	.45
		Seq vs. seq	.18	Seq	11	.15	Seq	13	1.24	.14	10.83	.20	.84
Absent													
No ID													
Cutler and Fisher (1990)	Live	—	.85	Sim	26	.57	Sim	21	4.27	1.08	17.00	2.06	.04
Kerstholt et al. (2004)	Live	—	.43	Sim	58	.71	Both	45	3.23	1.43	7.69	2.79	.01
Peters (1991)		Crime	.67	Sim	12	.42	Sim	12	2.80	.53	14.77	1.22	.22
		No crime	.50	Sim	12	.33	Sim	12	2.03	.39	10.59	.84	.40
Sporer (1991)	Live	Sim vs. seq	.31	Sim	13	.80	Seq	15	9.09	1.59	50.00	2.48	.01
		Seq vs. seq	.67	Seq	12	.80	Seq	15	1.96	.34	11.11	.76	.45
Both													
Choosing													
Cutler and Fisher (1990)	Live	—	.25	Sim	26	.41	Sim	21	2.08	.60	7.14	1.16	.25
Dent and Stephenson (1979) Exp 1	Live	—	.29	Sim	35	.49	Seq	35	2.33	.88	6.25	1.70	.09
Dent and Stephenson (1979) Exp 2	Live	—	.46	Sim	98	.61	Seq	124	1.85	1.08	3.13	2.22	.03
Dent and Stephenson (1979) Exp 4	Live	Live with screen	.70	Sim	50	.50	Seq	50	2.33	1.03	5.30	2.02	.04
		No screen	.36	Sim	50	.50	Seq	50	1.79	.80	4.00	1.41	.16
Peters (1991)	Live	Crime	.38	Sim	24	.75	Sim	24	5.00	1.43	16.67	2.51	.01
		No crime	.67	Sim	24	.83	Sim	24	2.38	.61	9.09	1.26	.21
Sporer (1991)	Live	Sim vs. seq	.75	Sim	28	.46	Seq	28	3.52	1.13	10.94	2.18	.03
		Seq vs. seq	.57	Seq	23	.46	Seq	28	1.56	.51	4.73	.78	.44
*Note*. *OR* = odds ratio; CI = confidence interval; LL = lower limit 95% CI; UL = upper limit 95% CI; Sim = simultaneous presentation; Seq = sequential presentation; BW = black and white (monochrome); ID = identification. Choosing rates are collapsed across target-present and target-absent or (if no target-absent lineups were used) target-present only. To facilitate comparisons, all *OR*s below 1.00 and associated CIs are converted to their inverse.													

**Table 3 tbl3:** Comparisons Between Photo and U.K. Video Lineups

Target, outcome, and study	Event	Condition	Photo			U.K. video			Effect size and CIs			Test of null	
			Rate	Type	*n*	Rate	Type	*n*	*OR*	LL	UL	*z*	*p*
Present													
Hit													
Beresford and Blades (2006)	Video	Elimination	.44	Sim	43	.33	Seq	42	1.60	.66	3.85	1.04	.30
		Modified	.49	Sim	43	.45	Seq	42	1.17	.50	2.75	.37	.71
		Standard	.47	Sim	43	.50	Seq	44	1.12	.49	2.63	.28	.78
Darling, Valentine, and Memon (2008)	Live	Descript-match	.48	Seq	23	.42	Seq	24	1.27	.40	4.03	.41	.68
		Suspect-match	.54	Seq	26	.44	Seq	27	1.49	.51	4.41	.73	.47
Havard, Memon, Clifford, and Gabbert (2010)	Video	—	.58	Seq	55	.68	Seq	53	1.54	.70	3.33	1.07	.28
Seale-Carlisle and Mickes (2016)	Video	—	.39	Sim	571	.20	Seq	554	2.56	1.96	3.34	6.88	<.001
Valentine, Darling, and Memon (2007)	Live	Strict	.31	Seq	29	.42	Seq	24	1.61	.52	5.00	.83	.41
		U.K.	.61	Seq	28	.71	Seq	24	1.56	.49	5.00	.75	.45
Filler													
Beresford and Blades (2006)	Video	Elimination	.21	Sim	43	.19	Seq	42	1.13	.39	3.29	.23	.82
		Modified	.26	Sim	43	.24	Seq	42	1.11	.42	2.97	.21	.83
		Standard	.28	Sim	43	.34	Seq	44	1.33	.53	3.33	.60	.55
Darling et al. (2008)	Live	Descript-match	.09	Seq	23	.08	Seq	24	1.14	.15	8.86	.12	.90
		Suspect-match	.08	Seq	26	.04	Seq	27	2.09	.19	22.78	.60	.55
Havard et al. (2010)	Video	—	.20	Seq	55	.25	Seq	53	1.33	.54	3.33	.62	.53
Seale-Carlisle and Mickes (2016)	Video	—	.31	Sim	571	.50	Seq	554	2.22	1.75	2.86	6.45	<.001
Valentine et al. (2007)	Live	Strict	.14	Seq	29	.08	Seq	24	1.87	.31	11.44	.68	.50
		U.K.	.11	Seq	28	.08	Seq	24	1.42	.21	9.42	.36	.72
No ID													
Beresford and Blades (2006)	Video	Elimination	.35	Seq	43	.48	Seq	42	1.72	.72	4.17	1.21	.23
		Modified	.26	Seq	43	.31	Seq	42	1.28	.50	3.33	.51	.61
		Standard	.26	Seq	43	.16	Seq	44	1.84	.64	5.30	1.14	.26
Darling et al. (2008)	Live	Descript-Match	.44	Seq	23	.50	Seq	24	1.27	.40	4.00	.41	.68
		Suspect-Match	.39	Seq	26	.52	Seq	27	1.69	.57	5.00	.95	.34
Havard et al. (2010)	Video	—	.22	Seq	55	.08	Seq	53	3.24	1.00	10.55	1.95	.05
Seale-Carlisle and Mickes (2016)	Video	—	.31	Sim	571	.30	Seq	554	1.05	.81	1.35	.36	.72
Valentine et al. (2007)	Live	Strict	.55	Seq	29	.50	Seq	24	1.22	.41	3.61	.36	.72
		U.K.	.29	Seq	28	.21	Seq	24	1.54	.43	5.51	.66	.51
Absent													
No ID													
Beresford and Blades (2006)	Video	Elimination	.63	Sim	43	.50	Seq	42	1.70	.72	4.05	1.21	.23
		Modified	.51	Sim	43	.57	Seq	42	1.27	.54	3.03	.55	.58
		Standard	.26	Sim	43	.29	Seq	41	1.16	.45	3.03	.31	.76
Darling et al. (2008)	Live	Descript-match	.62	Seq	21	.86	Seq	22	3.70	.85	16.67	1.74	.08
		Suspect-match	.82	Seq	28	.79	Seq	29	1.21	.33	4.51	.29	.78
Havard et al. (2010)	Video	—	.42	Seq	53	.50	Seq	54	1.39	.65	2.94	.83	.41
Seale-Carlisle and Mickes (2016)	Video	—	.45	Sim	577	.30	Seq	503	1.91	1.48	2.46	5.04	<.001
Valentine et al. (2007)	Live	Strict	.83	Seq	23	.96	Seq	26	5.26	.54	51.00	1.52	.12
		U.K.	.67	Seq	24	.88	Seq	24	3.50	.80	15.34	1.73	.08
Both													
Choosing													
Beresford and Blades (2006)	Video	Elimination	.51	Sim	86	.51	Seq	84	1.00	.55	1.82	.00	1.00
		Modified	.62	Sim	86	.56	Seq	84	1.28	.69	2.37	.79	.43
		Standard	.74	Sim	86	.77	Seq	85	1.18	.58	2.38	.46	.65
Darling et al. (2008)	Live	Descript-match	.48	Seq	44	.33	Seq	46	1.87	.80	4.40	1.44	.15
		Suspect-match	.39	Seq	54	.34	Seq	56	1.24	.57	2.70	.54	.59
Havard et al. (2010)	Video	—	.70	Seq	108	.70	Seq	107	1.00	.56	1.79	.00	1.00
Seale-Carlisle and Mickes (2016)	Video	—	.62	Sim	1148	.70	Seq	1057	1.43	1.19	1.69	3.95	<.001
Valentine et al. (2007)	Live	Strict	.33	Seq	53	.26	Seq	50	1.40	.60	3.29	.78	.44
		U.K.	.54	Seq	52	.46	Seq	47	1.38	.62	3.04	.79	.43
*Note*. *OR* = odds ratio; CI = confidence interval; LL = lower limit 95% CI; UL = upper limit 95% CI; Sim = simultaneous presentation; Seq = sequential presentation; BW = black and white (monochrome); ID = identification. Choosing rates are collapsed across target-present and target-absent or (if no target-absent lineups were used) target-present only. To facilitate comparisons, all *OR*s below 1.00 and associated CIs are converted to their inverse.													

**Table 4 tbl4:** Delays in Archival Studies

Medium and archival study	*n*	Event-to-lineup delay
Live		
Ainsworth and King (1988)	52	38% = 1 week or less
Valentine, Pickering, and Darling (2003)	558	13% = Less than 1 month
		30% = 1–2 months
		26% = 2–3 months
		31% = more than 3 months
Horry, Halford, Brewer, Milne, and Bull (2014)	833	22% = Less than 1 month
		55% = 1–3 months
		23% = more than 3 months
Photo		
Behrman and Davey (2001)	284	55% = 1 week or less
Steblay (2011)	82	26% = 2 days or less
Wells, Steblay, and Dysart (2015)	494	Median = 2 weeks
Video		
Memon, Havard, Clifford, Gabbert, and Watt (2011)	1,044	15% = Less than 1 week
		36% = Less than 1 month
		15% = Less than 2 months
		15% = Less than 6 months
		15% = 6 months or more
Horry, Memon, Wright, and Milne (2012)	1,039	23% = 1 week or less
		12% = 1–2 weeks
		(Median = 31 days)

## Footnotes

1 Live lineups are otherwise known as a physical lineup, a corporeal lineup, an identity parade, or (simply) a lineup. Photo lineups are otherwise known as photoarrays, photospreads, photopacks, photoboards, or (in some parts of Australia) digiboards. Video lineups are otherwise known as a videotaped lineup or a video identity parade (the former usually refers to a recording of a live lineup, whereas the latter usually refers to sequential presentation of video clips of each lineup member).

2 None of following model policies/legislation indicate a preference between photo and live lineups: Bill Blackwood Law Enforcement Management Institute of Texas, 2014; California Commission on the Fair Administration of Justice, 2008; Florida Legislature, 2017; General Assembly of the State of Ohio, 2010; Georgia General Assembly, 2008; Massachusetts: Suffolk County Task Force on Eyewitness Evidence, 2004; and Northampton Police Department, 2005; New Jersey: Office of the Attorney General, 2001; Rhode Island: Task Force to Identify and Recommend Policies and Procedures to Improve the Accuracy of Eyewitness Identification, 2010; State of Wisconsin Office of the Attorney General, 2010; Virginia Department of Criminal Justice Services, 2014.

3 The policy on solicitors attending lineup procedures was revised in the 2017 revision of Code D. If a solicitor’s presence could deter or distract the eyewitness, identification officers now have the discretion to deny requests by solicitors to be present for the identification. Code D also states that identification procedures must be video recorded (with sound).
